# Illness and medical and other expenditures: observations from western and eastern China

**DOI:** 10.1186/s12913-015-0730-6

**Published:** 2015-02-20

**Authors:** Jing Zhang, Benchang Shia, Huangdi Yi, Shuangge Ma, Chi Ma

**Affiliations:** Department of Economics, School of Economics, Xiamen University, Xiamen, China; Department of Statistics, School of Economics, Xiamen University, Xiamen, China; Department of Statistics and Information Science, FuJen Catholic University, New Taipei City, Taiwan; Department of Biostatistics, School of Public Health, Yale University, 60 College ST, New Haven, CT 06520 USA; Humanities and Social Science College, Beijing Institute of Petrochemical Technology, 19 Qingyuan North Rd, Daxing, Beijing 102617 China

**Keywords:** Illness condition, Medical expenditure, Household expenditure, Cross-region difference, China

## Abstract

**Background:**

Illness and the medical expenditure that follows have a profound impact on the well-being of individuals and households. China is a huge country with significant regional differences. The goal of this study is to investigate the associations of illness and medical expenditure with other categories of household expenditures, with special attention paid to the differences in observations between the western and eastern regions.

**Methods:**

A survey was conducted in six major cities in China, three in the east and three in the west, in 2011. Data on demographics, illness conditions, and medical and other expenditures were collected from 12,515 households.

**Results:**

In the analysis of the associations of illness conditions and medical expenditure with demographics, multiple significant associations were observed, and there are differences between the eastern and western regions. In univariate analyses, illness conditions and medical expenditure were found as having significant associations with other categories of expenditures. In multivariate analyses adjusting for household and household head characteristics, few associations were observed, and there exist differences between the regions.

**Conclusions:**

This study has provided empirical evidence on the associations of illness/medical expenditure with demographics and with other categories of expenditures. Differences across regions were observed in multiple aspects. The reasons underlying such differences are worth investigating further.

## Background

Illness has a profound impact on people’s lives. It has a direct effect on health conditions, and the medical expenditure that follows can have a deep and long-lasting impact (especially financially) on the well-being of individuals and households [1-4]. Specifically, illness can directly change the spending behaviors of individuals. In addition, with a limited budget, individuals and households may have to modify expenditures on other categories (for example, basic consumption, education, recreation, etc.) to accommodate medical expenditure [5]. In the literature, there has been a large number of studies on the distributions of illness and medical expenditure and their associations [6,7]. Moreover, it is commonly agreed that illness and medical expenditure can affect other categories of expenditures. However, detailed empirical studies are still limited.

In the literature, studies that are the most relevant to the present one include a study conducted in rural Vietnam [8], which found that households experiencing inpatient treatment and higher levels of outpatient treatment had significantly lower consumptions of food, education, and production means. In a study conducted in rural Thailand [9], similar observations were made. In China, Wang and others [10] conducted a community-based survey in poor rural areas and found that medical expenditure caused a reduction in household investment in human and physical capital. A survey conducted in western China [11] found multiple, significant associations between illness (both chronic diseases and inpatient treatments) and medical expenditure and other types of household consumptions.

Similar to some of the aforementioned studies, this study is concerned with the associations of illness and medical expenditure with other categories of expenditures in China. What is different from some of the existing studies [11], however, is that special attention is paid to the differences between the western and eastern regions. China is a huge developing country, with the world’s largest population and significant regional differences [12,13]. In general, the western region is much less developed and less populated than the eastern region. Differences have been observed in economic development, availability and quality of health care, education, and even demographic characteristics such as age and gender (mainly due to a migration of young male workers). In the literature, there are studies comparing the east against the west in terms of the distributions of illness conditions and medical expenditure [14]. However, it is still unknown whether their associations with other categories of expenditures have regional differences. This study aims to fill this knowledge gap. Another difference between this study and some of the existing ones is that data were collected using a phone survey as opposed to being gathered from government databases. Data analyzed in this study may contain more detailed micro-information, which is not emphasized by government databases. Further, unlike some published studies [8-10], this study focuses on major cities and surrounding areas. China is currently experiencing the largest rural-to-urban migration in human history, making studies focused on major cities increasingly important. With the aforementioned differences taken into consideration, this study may provide additional insights into the financial consequences of illness beyond what has been established by the existing literature.

## Methods

### Data collection

**Figure 1:**
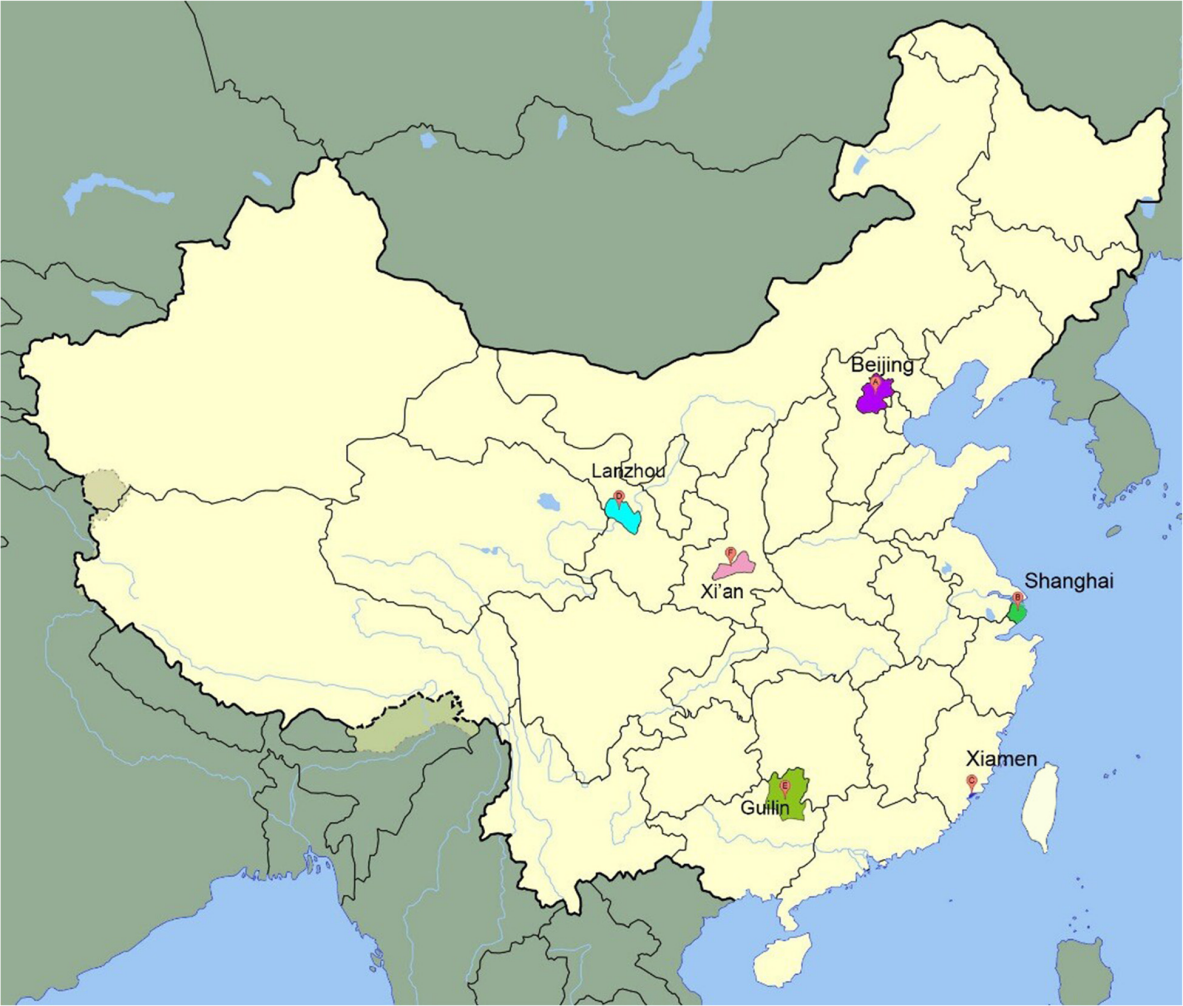
**Map of the six surveyed cities.**

This study was approved by a research ethics review committee at Xiamen University in China. A phone survey was then conducted in 2011. Six cities and their surrounding areas were selected for the survey. Among them, three are located in eastern China (Beijing, Shanghai, and Xiamen), and three are in western China (Lanzhou, Guilin, and Xi’an) (Figure 1). Beijing is the capital of China, with a population of 127.79 million and per capita GDP of 67,980 RMB in 2011 (NBS GDP Data [15]; 1 USD = 6.35 RMB at the time of the survey). Shanghai plays a major role in China’s economy. It had a population of 141.94 million and per capita GDP of 82,560 RMB in 2011. Xiamen is located on the southeast coast of the country and had a population of about 1.85 million in 2011 and per capita GDP of 70,734 RMB. Among the three western cities, Lanzhou is the capital city of the Gansu province. It had a population of 3.23 million and per capita GDP of 37,608 RMB in 2011. Guilin is in the Guangxi Zhuang Autonomous Region and had a population of 4.99 million in 2010 and per capita GDP of 27,843 RMB in 2011. Xi’an is the capital city of the Shanxi province and had a population of 7.92 million in 2011 and per capita GDP of 26,259 RMB. For comparison, the per capita GDP for the whole country of China was 35,198 RMB in 2011.

In conducting the survey, a computer-assisted telephone investigation system (CATI) was used. Candidate phone numbers were obtained from China Telecom Corp. Ltd. and Unicom Corp. The surveyed phone numbers were selected using an RDD (random-digit dialing) approach. Specifically, Mitofsky-Waksberg [16]-type samples of active blocks of 100 consecutive phone numbers were drawn from all such possible blocks within each city. For each block of phone numbers, the probability of being selected was proportional to the count of numbers serving residences. The database was updated after each phone call to ensure that no household was sampled multiple times. As it was difficult to associate a cell phone number with a physical location, the survey was limited to landlines only.

In China, households remain the main functional units for expenditure. Thus, all data collection and analysis were conducted at the household level. At the beginning of each survey, information was collected to determine inclusion. A household would be excluded if (a) the interviewee refused to participate, (b) the household was not officially registered in the surveyed city as defined by “hukou,” which is a household registration issued by the central government, (c) the interviewee was younger than 18 years old, or (d) the interviewee could not provide reliable information on the household. Verbal consent was obtained for each survey and recorded using voice-recording software. The survey included both snapshot questions (for example, demographic information and insurance status) and accumulative questions (for example, income and expenditure for a period of twelve months prior to the survey). On average, one survey took 8.5 minutes. The survey response rates were 39% and 42% in the western and eastern regions, respectively.

### Statistical analysis

An exploratory analysis was conducted first. No obviously unreasonable observation or outlier was found. A major focus of this study is to examine if there are any regional differences between the eastern and western regions. To this end, the analysis was conducted for the eastern and western regions separately and then compared. This strategy is equivalent to a model with full interaction between regions and other variables and is more flexible than simply including the region as a confounder in an additive model. The distributions of illness conditions, measured using the presence of chronic disease and inpatient treatment as well as medical expenditure over a period of twelve months, were first examined. Here, medical expenditure is the out-of-pocket expense defined as the gross expense minus insurance reimbursement. This better measures the real burden on households. It is a continuous measure. For presentational clarity, we dichotomized medical expenditure around the median and created the high and low expenditure groups with about-equal sizes. The associations of illness and medical expenditure with demographic variables were also examined. T-tests were used for continuous variables, and chi-squared tests were used for categorical variables. Multivariate regression analyses were conducted, examining the associations between illness conditions and household expenditure patterns. To obtain more detailed information, eight categories of expenditures were considered—basic expenditure (food, produce, etc.), education, savings/investment, entertainment, insurance, durable goods, alcohol/tobacco, and other. To account for different household sizes, per capita expenditure was calculated and analyzed. In regression, the adjusted confounders include household characteristics (size, presence of member(s) younger than 18, presence of member(s) older than 65, percentage of household members covered by basic health insurance, percentage of household members covered by commercial health insurance, city, hukou, and per capita income) and household head characteristics (age, gender, education, occupation, and marital status). Here, two sets of analyses were conducted. The first set analyzed the actual amount of expenditure (in RMB), and the second analyzed the percentage of each category of expenditure (as of the total expenditure), which may better describe the scenario with a fixed total budget, as the sum of percentages is one. Multivariate analyses were also conducted, investigating the associations between medical expenditure and other categories of expenditures.

## Results

This study collected data on 12,515 households (Table 1). The sample size was decided based on resource availability. Among the surveyed households, 46.84% had at least one member with a chronic disease (or multiple chronic diseases), and 58.81% had at least one episode of inpatient treatment. In the examination of the distributions of illness and medical expenditure, across the three eastern cities, the presence of chronic disease was differently distributed. In Beijing, 64.35% of the households had at least one member with a chronic disease, compared to 40.44% in Xiamen. The distribution of medical expenditure is also different across cities. Shanghai had the highest percentage of households in the high medical expenditure category (59.84%), compared to 33.56% for Xiamen. Across the three western cities, the distributions of the presence of inpatient treatment and medical expenditure are significantly different. In Lanzhou, 59.88% of the households had at least one inpatient treatment, compared to 56.20% for Xi’an. Xi’an had the highest percentage of households in the high medical expenditure group (54.57%). Differences across cities were also observed. However, as we are mainly interested in comparing the eastern region against the western region overall, in what follows, we combine cities in each region and do not report city-wide analysis results. City is included as a confounder in multivariate analysis.

**Table 1:** **Sample characteristics for the six cities**

			**Presence of chronic disease**		**Presence of inpatient treatment**		**Medical expenditure**	
		**Whole cohort**	**Yes**	**No**	**Yes**	**No**	**High**	**Low**
**Total sample**		12515	5862	6653	7360	5155	6388	6127
	*		46.84	53.16	58.81	41.19	51.04	48.96
**Eastern**	Beijing	2143	1379	764	1275	868	1173	970
	*		64.35	35.65	59.50	40.50	54.74	45.26
	Shanghai	2139	959	1180	1264	875	1280	859
	*		44.83	55.17	59.09	40.91	59.84	40.16
	Xiamen	1746	706	1040	1049	697	586	1160
	*		40.44	59.56	60.08	39.92	33.56	66.44
	p-value		(<0.001)		(0.823)		(<0.001)	
**Western**	Lanzhou	2151	972	1179	1288	863	1026	1125
	*		45.19	54.81	59.88	40.12	47.70	52.30
	Guilin	2128	912	1216	1243	885	1118	1010
	*		42.86	57.14	58.41	41.59	52.54	47.46
	Xi'an	2208	934	1274	1241	967	1205	1003
	*		42.30	57.70	56.20	43.80	54.57	45.43
	p-value		(0.126)		(0.047)		(<0.001)	

*Percentages.

### Associations of illness and medical expenditure with demographics

Results are shown in Table 2. Summary statistics are provided for the whole cohort. In the examination of the associations between the presence of chronic disease and demographics, hukou is significantly associated in the eastern region but not the western region. Specifically in the eastern region, among households with the presence of chronic disease, 34.26% were rural, compared to 38.77% of those without chronic disease. For both the eastern and western regions, income is significantly associated with the presence of chronic disease. It is interesting to note that, in the eastern region, households with the presence of chronic disease had a higher per capita income (14,913 RMB vs. 14,277 RMB). In contrast, in the western region, households without chronic disease had a higher per capita income (6,940 RMB vs. 6,680 RMB). Occupation is significantly associated with the presence of chronic disease in the western region but not the eastern region. In the examination of the associations between the presence of inpatient treatment and demographics, only the gender of the household head is significantly associated in the western region. Specifically, in the group with the presence of inpatient treatment, 76.83% of the household heads were male, compared to 74.48% in the group without inpatient treatment. More demographic factors are significantly associated with the level of medical expenditure in particular, including household size, presence of members older than 65, per capita income, and occupation. All four factors are significant for both the eastern and western regions. In addition, the “directions” (positive or negative associations) are the same.

**Table 2:** **Sample characteristics for the whole cohort and subgroups with different disease and treatment status and medical expense levels**

			**Presence of chronic disease**				**Presence of inpatient treatment**				**Medical expense**			
		**Whole cohort**	**Eastern**		**Western**		**Eastern**		**Western**		**Eastern**		**Western**	
			**Yes**	**No**	**Yes**	**No**	**Yes**	**No**	**Yes**	**No**	**High**	**Low**	**High**	**Low**
**Data on household**														
**Household size**		4.64	4.61	4.62	4.69	4.64	4.61	4.61	4.67	4.64	4.66	4.56	4.78	4.53
		(1.25)	(1.3)	(1.23)	(1.22)	(1.25)	(1.26)	(1.27)	(1.24)	(1.23)	(1.28)	(1.24)	(1.29)	(1.17)
p-value			(0.708)		(0.133)		(0.869)		(0.371)		(0.002)		(<0.001)	
Younger than 18*		0.36	0.33	0.32	0.4	0.4	0.32	0.33	0.40	0.40	0.32	0.32	0.4	0.4
p-value			(0.139)		(0.851)		(0.247)		(0.758)		(0.486)		(0.940)	
Older than 65*		0.34	0.37	0.38	0.31	0.31	0.37	0.37	0.31	0.31	0.38	0.36	0.32	0.30
p-value			(0.130)		(0.912)		(0.527)		(0.635)		(<0.001)		(<0.001)	
Hukou*	Urban	64.19	65.74	61.23	65.08	64.62	63.35	63.73	64.87	64.75	64.66	62.33	65.36	64.24
	Rural	35.81	34.26	38.77	34.92	35.38	36.65	36.27	35.13	35.25	35.34	37.67	34.64	35.76
p-value			(<0.001)		(0.721)		(0.785)		(0.941)		(0.064)		(0.360)	
**Income**		10570	14913	14277	6680	6940	14589	14611	6811	6850	21149	7938	9839	3613
		(8635)	(10030)	(9767)	(4883)	(4838)	(10010)	(9750)	(4888)	(4819)	(8299)	(6313)	(4470)	(2733)
p-value			(0.013)		(0.033)		(0.934)		(0.751)		(<0.001)		(<0.001)	
**Data on household head**														
Age*	<20	1.97	1.94	2.11	1.81	1.99	1.78	2.38	1.75	2.14	2.24	1.81	1.73	2.1
	21-30	8.24	8.28	8.04	7.95	8.59	8.08	8.28	8.7	7.77	8.56	7.76	8.84	8.38
	31-40	2.95	2.99	2.68	3.23	2.92	2.7	3.03	2.86	3.31	2.63	3.04	3.17	2.93
	41-50	36.78	37.12	36.19	37.47	36.44	36.65	36.68	36.32	37.68	36.89	36.43	36.97	36.81
	51-60	39.33	38.86	41.49	37.9	39.06	40.25	40.04	38.6	38.49	39.85	40.48	38.31	38.81
	>60	10.74	10.81	9.48	11.64	11.01	10.54	9.59	11.77	10.61	9.84	10.47	11.59	10.96
p-value			(0.272)		(0.680)		(0.488)		(0.252)		(0.510)		(0.825)	
Gender*	Male	76.52	76.87	77.65	76.19	75.58	77.37	77.09	76.83	74.48	77.72	76.78	76.23	75.43
p-value			(0.492)		(0.590)		(0.824)		(0.031)		(0.400)		(0.469)	
Education*	<middle school	49.47	49.61	49.4	49.04	49.74	48.63	50.78	49.15	49.83	49.39	49.62	49.3	49.59
	High school	39.34	39.75	39.61	39.21	38.89	40.64	38.28	39.45	38.45	39.68	39.68	38.28	39.87
	Bachelor	9.36	8.8	9.31	9.65	9.62	9.03	9.1	9.31	10.09	9.05	9.07	10.39	8.83
	>Bachelor	1.83	1.84	1.68	2.09	1.74	1.7	1.84	2.09	1.62	1.88	1.64	2.06	1.72
p-value			(0.876)		(0.747)		(0.313)		(0.330)		(0.920)		(0.102)	
Marital status*	Single	23.68	23.82	22.96	24.2	23.77	22.6	24.55	24.31	23.46	23.43	23.35	24.13	23.77
	Married	62.7	61.5	63.17	62.95	63.12	63.1	61.19	63.1	62.98	62.75	61.89	63.09	63
	Divorced	7.18	8.02	7.71	6.53	6.57	7.83	7.91	6.12	7.15	7.57	8.16	6.72	6.37
	Widowed	6.43	6.67	6.17	6.32	6.54	6.47	6.35	6.47	6.41	6.25	6.59	6.06	6.85
p-value			(0.587)		(0.967)		(0.356)		(0.392)		(0.770)		(0.583)	
Occupation*	Government	16.25	16.23	14.95	16.36	17.25	15.61	15.57	16.41	17.5	23.96	7.09	27.17	5.86
	State-owned Co.	21.73	21.91	21.45	20.83	22.51	21.35	22.17	21.5	22.17	34.52	8.63	37.44	5.07
	Private Co.	5.58	5.72	5.9	5.22	5.48	6.33	5.04	5.59	5.05	10.27	1.27	9.94	0.48
	Self-employed	19.42	19.48	19.47	20.87	18.23	19.65	19.22	19.99	18.53	0.3	38.98	1.7	38.24
	Farmer	31.32	30.85	3.77	30.2	31.4	31.49	32.25	30.91	30.83	30.87	32.75	23.08	39.2
	Unemployed	1.72	1.74	1.78	1.74	1.64	1.73	1.8	1.67	1.69	0.03	3.51	0.18	3.28
	Retired	2.95	2.96	2.65	3.62	2.67	2.81	2.79	2.97	3.24	0	5.65	0.42	5.93
	Other	1.02	1.12	1.04	1.17	0.82	1.03	1.15	0.95	0.99	0.07	2.11	0.06	1.94
p-value			(0.757)		(0.023)		(0.633)		(0.724)		(<0.001)		(<0.001)	

Mean (standard deviation) or percentage (variables marked by *).

### Associations between illness conditions and expenditure

In the whole cohort, the biggest expenditure category is basic expenditure (per capita 4,164.6 RMB), which includes food, production means, etc. The second-largest category is savings and investment (per capita 3,123.1 RMB), followed by insurance (per capita 2,050.4 RMB). Other categories are relatively small. More detailed results are presented in Table 3.

**Table 3:** **Household expenditure for the whole cohort and subgroups with different disease and treatment status and medical expense levels**

		**Presence of chronic disease**				**Presence of inpatient treatment**				**Medical expenditure**			
		**Eastern**		**Western**		**Eastern**		**Western**		**Eastern**		**Western**	
	**Whole cohort**	**Yes**	**No**	**Yes**	**No**	**Yes**	**No**	**Yes**	**No**	**High**	**Low**	**High**	**Low**
**Amount of expenditure (RMB)**													
Basic	4164.6	5886.9	5639.1	2580.9	2753	5780.6	5740.1	2656	2709.2	8532.1	2950.1	4039	1226
sd	3801.3	4455.2	4435.7	2131.5	2229.1	4512.3	4388.1	2190.3	2186.7	4183.8	2578.7	2135.6	975.4
p-value		(0.031)		(0.002)		(0.728)		(0.334)		(<0.001)		(<0.001)	
Education	668.8	990.3	947.8	417.1	436.7	965.6	974.6	426.9	429.9	1451.4	479	666.5	173.8
sd	624.4	734.8	709.4	352.6	356.3	723.6	721.2	360	347.7	622.2	427.4	318.4	163.7
p-value		(0.022)		(0.028)		(0.636)		(0.738)		(<0.001)		(<0.001)	
Savings/Investment	3123.1	4395.7	4243.7	1955.6	2052.6	4325.4	4313.2	204.6	2018.7	6403.3	2202.7	3042.1	909.5
sd	2723.8	3189.4	3135.5	1543.3	1577.7	3211.5	3092.1	1592.1	1522.9	2795.1	1828.6	1445.5	689.4
p-value		(0.062)		(0.013)		(0.882)		(0.718)		(<0.001)		(<0.001)	
Entertainment	688.1	981.2	951.2	416.1	439.8	965.8	967.1	426.7	433.4	1448	476.6	667.4	175.6
sd	621.3	720.1	714.3	351.5	361.7	726.8	703.3	361.3	352	612.2	425.4	323.7	165.6
p-value		(0.104)		(0.008)		(0.943)		(0.456)		(<0.001)		(<0.001)	
Insurance	2050.4	2906.2	2795.8	1276.2	1328.9	2842.4	2865	1289	1329.7	4224.3	1455.9	1981.7	585
sd	1882.2	2229.1	2185.1	1065.4	1080.9	2232.2	2172.2	1071.9	1077.7	2061.7	1283.2	1032.9	490.7
p-value		(0.052)		(0.050)		(0.696)		(0.133)		(<0.001)		(<0.001)	
Durable goods	374.9	547.1	519.5	219.4	234	531.9	535.6	224.3	232.3	810.8	251.4	364.4	81.7
sd	378.7	445.5	439.8	216.9	226	441.7	444.7	222.4	221.8	407.6	262.4	218.8	98.4
p-value		(0.015)		(0.009)		(0.752)		(0.154)		(<0.001)		(<0.001)	
Alcohol/Tobacco	165	247.8	240	88.8	93.9	244.2	243.6	91.4	92.2	380.3	105.4	155.6	23.6
sd	189.6	223.7	223.2	107.3	109.7	226.1	219.6	109.8	107.2	213.4	127.7	111.3	46.9
p-value		(0.178)		(0.060)		(0.909)		(0.760)		(<0.001)		(<0.001)	
Other	10.2	14.3	16.3	6.4	4.7	13.4	18	5.4	5.5	17.6	12.8	6	4.9
sd	115.7	148.3	161.5	65.8	53.1	139.6	175.1	52.5	66.9	181.4	122.2	66	50.3
p-value		(0.623)		(0.271)		(0.287)		(0.926)		(0.229)		(0.450)	
**Percentage of expenditure**													
Basic	31.26	31.20	31.07	31.26	31.69	31.18	31.06	31.47	31.56	30.69	32.51	31.01	33.42
Education	5.17	5.25	5.22	5.05	5.03	5.21	5.27	5.06	5.01	5.22	5.28	5.12	4.74
Savings/Investment	23.44	23.30	23.38	23.68	23.63	23.33	23.34	23.76	23.51	23.03	24.27	23.35	24.79
Entertainment	5.16	5.20	5.24	5.04	5.06	5.21	5.23	5.06	5.05	5.21	5.25	5.12	4.79
Insurance	15.39	15.40	15.40	15.46	15.30	15.33	15.50	15.28	15.49	15.2	16.04	15.21	15.95
Durable goods	2.81	2.90	2.86	2.66	2.69	2.87	2.90	2.66	2.71	2.92	2.77	2.80	2.23
Alcohol/Tobacco	1.24	1.31	1.32	1.08	1.08	1.32	1.31	1.08	1.07	1.37	1.16	1.19	0.64
Other	0.08	0.08	0.09	0.08	0.05	0.07	0.10	0.06	0.06	0.06	0.14	0.05	0.13

The marginal association analysis results are presented in Table 3. For both the eastern and western regions, basic, education, and durable goods expenditures were found to have significant associations with the presence of chronic disease. It is interesting to note that the “directions” of associations are different. Specifically, for all three categories of expenditure, in the eastern region, the group with chronic disease had a higher level, whereas in the western region, the group without chronic disease had a higher level. In addition, in the western region, the presence of chronic disease was also significantly associated with savings/investment, entertainment, and insurance. The presence of inpatient treatment was not significantly associated with any category of expenditure in the eastern or western regions. The level of medical expenditure was found to be significantly associated with seven categories of expenditures (all except for “other”). Specifically, for both the eastern and western regions and for all seven categories, the level of medical expenditure had positive associations.

For illness conditions, the first set of multivariate analyses were conducted on the amount of expenditure. The analysis results, including the estimated regression coefficients and corresponding p-values, are shown in Table 4. Judged by the p-values, the presence of chronic disease was not significantly associated with expenditure, after adjusting for the confounders. In the eastern region, the presence of inpatient treatment was not significantly associated with expenditure. In the western region, the presence of inpatient treatment was found to be significantly negatively associated with insurance (estimated regression coefficient of −34.49 RMB) and durable goods (estimated regression coefficient of −6.64 RMB).

**Table 4:** **Multivariate linear regression analysis: effects of presence of chronic diseases and inpatient treatments on expenditure (actual amount in RMB)**

	**Presence of chronic disease**				**Presence of inpatient treatment**			
	**Eastern**		**Western**		**Eastern**		**Western**	
	**Est.**	**p-value**	**Est.**	**p-value**	**Est.**	**p-value**	**Est.**	**p-value**
Basic	14.23	0.808	−59.26	0.062	48.60	0.412	−38.44	0.223
Education	2.38	0.735	−0.15	0.971	−8.72	0.221	−0.53	0.901
Medical expense	−10.26	0.731	9.67	0.548	44.87	0.138	−13.16	0.416
Savings/Investment	−20.79	0.534	−13.40	0.491	8.84	0.794	−4.26	0.827
Entertainment	−10.07	0.158	−4.30	0.319	−0.06	0.993	−4.32	0.319
Insurance	−2.43	0.935	1.90	0.901	−20.45	0.499	−34.49	0.025
Durable goods	6.68	0.261	−3.21	0.322	−2.94	0.625	−6.64	0.041
Alcohol/Tobacco	−4.31	0.156	−0.17	0.920	0.76	0.806	−0.29	0.867

Est.: estimated regression coefficient in multivariate linear regression, adjusted for household characteristics (size, presence of younger than 18, presence of older than 65, percentage of basic insurance, percentage of commercial insurance, city, urban, income) and household head characteristics (age, gender, education, occupation, marital status).

The second set of multivariate regression analyses were conducted on the percentage of each category of expenditure (defined as the ratio of the amount of expenditure in a specific category over the total expenditure).The results are shown in Table 5. In the eastern region, the presence of chronic disease was not associated with any expenditure. In contrast, in the western region, it was found to be significantly associated with a decrease in basic expenditure (aOR = 0.975) and an increase in insurance (aOR = 1.026). The presence of inpatient treatment was not associated with any expenditure in the eastern region. In comparison, in the western region, it was significantly associated with an increase in education (aOR = 1.018) and a decrease in insurance (aOR = 0.973).

**Table 5:** **Multivariate logistic regression analysis: effects of the presence of chronic diseases and inpatient treatments on expenditure (percentage as of total expenditure)**

	**Presence of chronic disease**				**Presence of inpatient treatment**			
	**Eastern**		**Western**		**Eastern**		**Western**	
	**aOR**	**p-value**	**aOR**	**p-value**	**aOR**	**p-value**	**aOR**	**p-value**
Basic	1.013	0.184	0.975	0.028	1.009	0.388	0.995	0.675
Education	1.001	0.824	1.009	0.237	0.988	0.077	1.018	0.022
Medical expense	0.992	0.457	1.013	0.280	1.017	0.092	1.005	0.688
Savings/Investment	0.993	0.341	0.995	0.842	1.000	0.968	1.011	0.180
Entertainment	0.993	0.284	1.000	0.963	0.994	0.347	1.006	0.442
Insurance	1.000	0.984	1.026	0.029	0.985	0.134	0.973	0.019
Durable goods	1.015	0.162	1.004	0.762	0.992	0.465	0.981	0.157
Alcohol/Tobacco	0.979	0.072	1.004	0.717	1.006	0.611	1.018	0.156

aOR: adjusted odds ratio in multivariate logistic regression, adjusted for household characteristics (size, presence of younger than 18, presence of older than 65, percentage of basic insurance, percentage of commercial insurance, city, urban, income) and household head characteristics (age, gender, education, occupation, marital status).

### Associations between medical expenditure and other expenditures

The analysis results on the effects of medical expenditure on the percentages of other expenditures (as of total expenditure) are shown in Table 6. For both the eastern and western regions, medical expenditure was found to be negatively associated with basic expenditure (aOR = 0.689 and 0.664), savings/investment (aOR = 0.886 and 0.901), and insurance (aOR = 0.841 and 0.859). In addition, medical expenditure had a significant negative association with expenditure on durable goods in the eastern region (aOR = 0.953).

**Table 6:** **Multivariate logistic-type regression analysis: impact of medical expenditure on other household expenditures**

	**Percentage of medical expenditure**			
	**Eastern**		**Western**	
	**aOR**	**p-value**	**aOR**	**p-value**
Basic	0.689	<0.001	0.664	<0.001
Education	0.983	0.083	0.989	0.301
Savings/Investment	0.886	<0.001	0.901	<0.001
Entertainment	0.992	0.403	0.986	0.219
Insurance	0.841	<0.001	0.859	<0.001
Durable goods	0.953	0.003	0.981	0.315
Alcohol/Tobacco	1.005	0.777	0.988	0.482

aOR: adjusted odds ratio in multivariate logistic regression, adjusted for household characteristics (size, presence of younger than 18, presence of older than 65, percentage of basic insurance, percentage of commercial insurance, city, urban, income) and household head characteristics (age, education, occupation, and marital status.

## Discussion

According to public data [17], in 2011 in the Gansu province (where Lanzhou is located), the per capita income was 14,989 RMB for urban areas and 3,909 RMB for rural areas. In the Guangxi Zhuang Autonomous Region (Guilin), the per capita income was 18,854 RMB for urban areas and 5,231 RMB for rural areas. In the Shanxi province (Xi’an), the per capita income was 18,245 RMB for urban areas and 5,028 RMB for rural areas. In the Fujian province (Xiamen), the per capita income was 33,565 RMB for urban areas and 11,928 RMB for rural areas. For the whole surveyed cohort, the per capita income was 10,570 RMB (sd 8,635 RMB). The income data suggest that the surveyed households might provide sensible information for their corresponding provinces. In addition, all six cities have large populations and play important economic and political roles in the country. Thus, even with the limited sample collection, this study is still of considerable value.

Within each region, cross-city differences in the distributions of illness conditions and medical expense were observed. As seen in the income data, the cities have significantly different economic statuses. In addition, as seen in the downstream analyses, illness conditions and medical expense are associated with multiple demographic and other factors. Moreover, many other factors (for example, geography, food consumption, and culture) may also contribute to the differences across cities. As cross-city difference was not the main focus of this study, it was not investigated further.

In the association analysis of illness conditions with demographics, the findings are mostly positive in the sense that we did not identify many personal factors that make certain individuals/households more susceptible to diseases. For the presence of chronic disease, hukou is significant in the eastern region but not the western region. In the eastern region, overall economic status is high, and the rural–urban difference (in the economy and other aspects) is less than that of the western region. It is, thus, reasonable to observe more households without chronic disease in the rural areas of the eastern region. The presence of chronic disease is positively associated with income in the eastern region but negatively associated in the western region. It has been noted that, in the relatively wealthy areas of China, increased income has been associated with significant increases in, for example, obesity, cardiovascular diseases, diabetes, cancers, and other diseases. In contrast, in the less wealthy areas, the presence of chronic disease may lead to reduced working capability and, hence, lower income. Occupation can be confounded with many other factors, including, for example, age, education, socioeconomic status, insurance status, etc. This may partly explain its association with the presence of chronic disease. A similar argument may hold for the association between the presence of inpatient treatment and the gender of the head of the household. The associations between medical expense and demographics have been noted in multiple publications [18-20]. For the eastern and western regions of China, the observed magnitudes were different, which is partly attributable to the difference in socioeconomic status between the regions. However, the “directions” are the same, suggesting that the two regions follow similar mechanisms.

In the univariate analysis, the presence of chronic disease was significantly associated with multiple categories of expenditures. However, we observed different “directions” for basic expenditure, education, and durable goods. Expenditure overall and for each category was significantly associated with income and socioeconomic status. The observed reversed patterns are possibly reasonable, given the discussion on income presented above. Another observation is that the variances of expenditures are large. A closer examination of the data suggests that there is a proportion of observations with very high expenditure levels. Ideally, a stratified analysis or robust analysis can be conducted to accommodate those large values. We chose standard deviation (as a summary statistic) and standard regression analysis for interpretation and sample size considerations. It is “relieving” to observe that there is no significant association between the presence of inpatient treatment and expenditure, indicating that such health shocks may have limited impact on the surveyed households.

In the multivariate analysis, after accounting for the effects of multiple household and household head characteristics, there was almost no association between illness and expenditure. In the western region, the presence of inpatient treatment led to decreased expenditure on insurance and durable goods. From this, very intuitive interpretations can be made. It is noted that the estimated magnitudes are small, which is in accordance with published studies [11]. The lack of association suggests that the surveyed households were well protected from health shocks. This observation is different from those mentioned in the background section of this paper. The difference can be caused by the significantly higher economic status and higher health insurance coverage of the surveyed cities, as well as the constantly improving health care system in China in the recent years, especially since the health care and health insurance reform of 2009. Different observations were also made in the analysis of the percentage of expenditure. This set of analyses mimics the fixed and same-budget scenario, which is different from the previous analysis. Chronic disease and inpatient treatment represent different types of health shocks [4,21,22] and, hence, have different impacts. The analysis suggests that households in the eastern region may be better protected in the sense that the expenditure structure is not significantly affected by illness. In the eastern region, the negative association for basic expenditure and positive association for insurance have intuitive interpretations. The reduction in basic expenditure is particularly worth noting. Education makes up a relatively small percentage of expenditure. The observed association needs to be interpreted with caution. Medical expense leads to decreases in the percentages of multiple categories of expenditures. Considering the fixed sum of percentages, such an observation is very sensible. We observed that the eastern and western regions follow similar patterns.

### Limitations

This survey collected data from six cities, with three in each region. We do not expect the six cities to be able to represent the whole country of China. Observations made in this study may be extendable to cities with similar socioeconomic and health conditions. More extensive sample collection was not conducted because of resource limitation. Only cross-sectional, observational data were collected. Such data have limitations. For example, no causal effect can be deduced. In addition, only a small amount of information was collected because of the phone call nature of the survey. For example, the complex illness condition is only represented using two variables. It is inevitable that some important variables may have been missed in the data collection. The chosen set of selected variables was motivated by multiple published studies and may be of the most importance. Interviewees were asked to recall information for a period of twelve months to reduce seasonality effects. Such an approach may have a recall bias [23]. This problem is potentially shared by multiple publications. The analysis provides empirical evidence on the differences between the eastern and western regions of China. Possible explanations based on health and socioeconomic and personal factors are provided. Further information collection is needed to draw more affirmative conclusions. For example, information on policy is not available in this study. It has been noted that health care and health insurance policies vary across regions and between rural and urban areas. Management factors are also not accounted for.

## Conclusions

The impact of illness and medical expenditure on other categories of expenditures has been acknowledged. This study has described a phone survey and conducted empirical study of such an impact. There is an emphasis on regional differences. The development of the economy and health care in the western region has been lagging behind. This study suggests that illness may also affect expenditure in different manners.
